# An Exploration of Discrepant Recalls Between AI and Human Readers of Malignant Lesions in Digital Mammography Screening

**DOI:** 10.3390/diagnostics15121566

**Published:** 2025-06-19

**Authors:** Suzanne L. van Winkel, Ioannis Sechopoulos, Alejandro Rodríguez-Ruiz, Wouter J. H. Veldkamp, Gisella Gennaro, Margarita Chevalier, Thomas H. Helbich, Tianyu Zhang, Matthew G. Wallis, Ritse M. Mann

**Affiliations:** 1 Radboudumc—Radboud University Nijmegen Medical Centre, Medical Imaging Department, Geert Grooteplein Zuid 10, 6525 GA Nijmegen, The Netherlands; ioannis.sechopoulos@radboudumc.nl (I.S.); tianyu.zhang@radboudumc.nl (T.Z.); ritse.mann@radboudumc.nl (R.M.M.); 2 Amsterdam UMC, Location VUmc (Vrije Universiteit Medical Center), Faculty of Medicine, Department of Ethics, Law & Humanities, De Boelelaan 1089a, 1081 HV Amsterdam, The Netherlands; 3 LRCB—Dutch Expert Centre for Screening, Wijchenseweg 101, 6538 SW Nijmegen, The Netherlands; 4 Technical Medicine Centre, University of Twente, Hallenweg 5, 7522 NH Enschede, The Netherlands; 5 ScreenPoint Medical BV, Toernooiveld 300, 6525 EC Nijmegen, The Netherlands; 6 Leiden University Medical Center, Department of Radiology, Albinusdreef 2, 2333 ZA Leiden, The Netherlands; 7 Veneto Institute of Oncology (IOV), Department of Imaging and Radiotherapy, Unit of Breast Radiology, IRCCS, via Gattamelata 64, 35128 Padua, Italy; gisella.gennaro@iov.veneto.it; 8 Department of Radiology and Medical Physics, Universidad Complutense de Madrid, 28040 Madrid, Spain; 9 Department of Biomedical Imaging and Image-Guided Therapy, Division of General and Pediatric Radiology, Medical University of Vienna and General Hospital, Waehringer Guertel 18-20, A-1090 Vienna, Austria; 10 Netherlands Cancer Institute, Antoni van Leeuwenhoek (NKI), Department of Radiology, Plesmanlaan 121, 1066 CX Amsterdam, The Netherlands; 11 Cambridge Breast Unit and NIHR Cambridge Biomedical Research Centre, Addenbrooke’s Hospital, Cambridge CB2 2QQ, UK; matthewwallis492@btinternet.com

**Keywords:** artificial intelligence, digital mammography, breast cancer detection, human vs. AI discrepancies, radiologist reader study

## Abstract

**Background:** The integration of artificial intelligence (AI) in digital mammography (DM) screening holds promise for early breast cancer detection, potentially enhancing accuracy and efficiency. However, AI performance is not identical to that of human observers. We aimed to identify common morphological image characteristics of true cancers that are missed by either AI or human screening when their interpretations are discrepant. **Methods:** Twenty-six breast cancer-positive cases, identified from a large retrospective multi-institutional digital mammography dataset based on discrepant AI and human interpretations, were included in a reader study. Ground truth was confirmed by histopathology or ≥1-year follow-up. Fourteen radiologists assessed lesion visibility, morphological features, and likelihood of malignancy. AI performance was evaluated using receiver operating characteristic (ROC) analysis and area under the curve (AUC). The reader study results were analyzed using interobserver agreement measures and descriptive statistics. **Results:** AI demonstrated high discriminative capability in the full dataset, with AUCs ranging from 0.903 (95% CI: 0.862–0.944) to 0.946 (95% CI: 0.896–0.996). Cancers missed by AI had a significantly smaller median size (9.0 mm, IQR 6.5–12.0) compared to those missed by human readers (21.0 mm, IQR 10.5–41.0) (*p* = 0.0014). Cancers in discrepant cases were often described as having ‘low visibility’, ‘indistinct margins’, or ‘irregular shape’. Calcifications were observed in 27% of human-missed cancers (42/154) versus 18% of AI-missed cancers (38/210). A very high likelihood of malignancy was assigned in 32.5% (50/154) of human-missed cancers compared to 19.5% (41/210) of AI-missed cancers. Overall inter-rater agreement was poor to fair (<0.40), indicating interpretation challenges of the selected images. Among the human-missed cancers, calcifications were more frequent (42/154; 27%) than among the AI-missed cancers (38/210; 18%) (*p* = 0.396). Furthermore, 50/154 (32.5%) human-missed cancers were deemed to have a very high likelihood of malignancy, compared to 41/210 (19.5%) AI-missed cancers (*p* = 0.8). Overall inter-rater agreement on the items assessed during the reader study was poor to fair (<0.40), suggesting that interpretation of the selected images was challenging. **Conclusions:** Lesions missed by AI were smaller and less often calcified than cancers missed by human readers. Cancers missed by AI tended to show lower levels of suspicion than those missed by human readers. While definitive conclusions are premature, the findings highlight the complementary roles of AI and human readers in mammographic interpretation.

## 1. Introduction

Breast cancer is a significant cause of mortality among women, ranking second in terms of cancer-related deaths [1]. Early diagnosis plays a critical role in increasing the chances of successful treatment [1,2]. Digital mammography (DM)-based screening enables the earlier detection of breast cancer (BC) and has been shown to reduce mortality by 20–35% among European women aged 50–69 [3].

To provide a consistent language for interpreting and reporting of mammographic findings, the American College of Radiology (ACR) introduced the Breast Imaging Reporting and Data System (BI-RADS) lexicon to describe specific morphological characteristics of the breast and lesions in the early 1990s [4,5]. BI-RADS provides standardized terminology to describe mammographic features, including breast composition, masses, asymmetries, architectural distortions, calcifications, and remaining associated findings [5]. Final assessment categories are assigned on a forced six-point scale (BI-RADS 1–6), describing the suspicion of malignancy, from negative findings (BI-RADS 1) to biopsy-proven cancer (BI-RADS 6).

Recently, there has been a notable advancement in artificial intelligence (AI) systems designed for the automated evaluation of screening mammograms, approaching the performance levels of human radiologists [6,7,8,9,10]. Considering the scarcity in some countries of skilled radiologists capable of accurately interpreting mammograms, AI has the potential to support current mammography-based screening by reducing the workload of human radiologists [10,11,12,13], as well as improving screening quality through reduced reading time and enhanced accuracy [7,10,14,15]. However, the integration of AI into clinical workflows raises challenges related to interpretability, trust, and optimal collaboration between AI and human readers.

Despite the increasing performance of AI systems for the automated evaluation of screening mammograms, even the most advanced ones occasionally make mistakes. At the same time, AI may detect lesions that are missed by human readers. AI-based image classification algorithms learn to identify lesion features through training, rather than relying on human-defined rules [16]. To minimize errors in screening and determine the most effective scenario in which human and AI screening interpretation can complement each other, it is crucial to understand why certain lesions are missed or misinterpreted as benign by AI. In addition, it is important to identify the image characteristics associated with a higher risk of missed diagnoses by human readers, particularly in cases where AI may produce more reliable results given the same characteristics.

Therefore, the objective of this study is twofold: first, to gain a better understanding of the challenges faced by AI in detecting breast cancers on mammography, and second, to assess whether certain image characteristics in cases with discrepant AI and human interpretations make it more likely that either AI or human interpretations are more credible.

By using a large retrospective dataset comprising digital mammography images, multiple observer interpretations, and verified true outcomes, we first analyzed the overall performance of a specific AI system in this particular retrospective dataset. Subsequently, we identified cases of breast cancer where human and AI results disagreed. These cases were then included in a reader study to evaluate the associated radiological features.

Through this study, we aim to identify common morphological image characteristics of cancers that are missed by either AI or human screening when their interpretations are discrepant.

## 2. Materials and Methods

### 2.1. Study Design

This study evaluates challenging cases of breast cancer in mammograms, in which AI and retrospectively obtained human interpretation results disagreed. These retrospectively obtained problematic screening mammograms were included in a prospective reader study to assess the possibility of identifying any common characteristics. To provide some context regarding the discriminative ability of the AI, a performance evaluation was also conducted.

### 2.2. Retrospective Screening Data

A subset of the dataset that was assembled for the study by Rodriguez Ruiz et al. (2019) [7] was retrieved for this study. The available data consists of digital mammography (DM) exams that were read by multiple radiologists during several other retrospective multi-reader multi-case observer studies [17,18,19,20,21,22,23], in which DM was compared to DBT for breast cancer detection in cancer-enriched datasets. The cases were obtained from six different institutions across Europe (Table 1). The review board at each involved institution approved the use of this data for retrospective research. In total, 1829 exams were included, with 9843 independent interpretations from 39 readers.

Each subset consisted of either unilateral or bilateral 2D DM exams, including cranio-caudal (CC) and medio-lateral oblique (MLO) views. In all datasets, the radiologists individually scored each screening exam, without time constraint or access to other imaging techniques (including any supporting AI systems).

Within some subsets, the radiologists had access to priors (which were not processed by the AI system under investigation). Table 1 shows the distribution of the radiologists’ experience with mammography for each subset: all readers were specialized in breast imaging and qualified according to the European breast cancer screening guidelines for quality assurance [24]. The human raters were instructed to score the way they would usually do in a screening setting. The corresponding radiologists’ interpretations for each DM exam were based on single readings by individual radiologists, resulting in either BI-RADS-scores or probability of malignancy (PoM) scores (scale 1–100%). In this study, any BI-RADS-score > 2 was considered a recall.

In one retrospective screening data subset (Subset B; N = 263), human ratings were only available in the form of PoMs (Table 1). Based on the average human reader recall rate in the remaining subsets (43.2%, considering BI-RADS > 2 as a recall), the associated PoM of >30 was interpreted as equal to BI-RADS > 2.

### 2.3. Ground Truth

The ground truth was available in the form of a dichotomized outcome regarding the presence of breast cancer at the case level, confirmed by histopathology or by the occurrence of a diagnosis within at least one year of follow-up.

### 2.4. Artificial Intelligence

The dataset was analyzed using a commercially available AI algorithm solution for mammography (Insight MMG, Lunit Inc., Seoul, Republic of Korea). The highest reported per breast suspicion score (0–100%) was used as the case-level AI score. The algorithm was developed based on deep convolutional neural networks (CNNs), including ResNet-34 [25,26]. The retrospectively obtained mammographic screens that were included for conducting this study were not involved in training or validation of the AI system under investigation.

Based on the advice of the vendor, all mammograms with a >10% AI score were handled as suspicious cases that would have been recalled by AI. In addition, the AI algorithm also provides a categorical breast density score according to the BI-RADS 5th edition [5] categories by averaging the MLO- and CC-view density levels per breast, applying the thresholds of < 3.5% (A), <7.5% (B), <15.5% (C), and ≥15.5% (D). The most dense category per breast was assigned for case-level analysis.

### 2.5. Case Categorization

Cases were categorized as an AI-missed cancer if breast cancer was diagnosed, at least one human reader in the retrospective studies rated the case as BI-RADS > 2, and the AI rated it as <10.00% breast cancer suspicion. Conversely, cases were categorized as human-reader-missed cancer if breast cancer was diagnosed, at least one human reader during the retrospective studies scored the case as BI-RADS < 3, and the AI rated it as ≥10.00%. These two groups represent discrepant interpretations between AI and human readers and form the basis of the comparative analyses presented in the following sections.

Since 8 four-view cases only had a case-level truth outcome available, both laterality views (two-view) were included in the reader study, allowing for a majority vote in order to retain only the laterality with true breast cancer for further analysis. A 60% vote threshold, combined with consistency in tumor location (image quadrant), determined which laterality to retain for analysis.

### 2.6. Online Reader Study

#### 2.6.1. Facilitating Software

Grand Challenge (Grand-Challenge.org v2023.05, Diagnostic Image Analysis Group; Radboud University Medical Center, The Netherlands) [27], an open-source software for performing reader studies that can be run online, was used for this study. The platform presents the user with the study images and a programmable set of questions, including the option to annotate.

For this study, following the BI-RADS 5th edition lexicon [5] topics, the reader study included seven multiple-choice questions related to breast composition, lesion visibility, lesion shape, breast density, and presence (none; typically benign; typically suspected of malignancy) of calcifications. Additionally, the raters were requested to annotate the image to mark the location of a visible breast lesion. The raters were also asked to provide a case-level PoM score (in a range of 0–100%). In addition, the raters were also asked to classify the likelihood of a malignancy on a forced 3-point scale (unlikely; uncertain; very likely), offering less freedom of choice.

The exact questions are included in the Supplementary Materials. The readers had no access to any additional patient information.

#### 2.6.2. Study Readers

A total of 28 readers, affiliated with hospitals from 20 different countries, were included in the online reader study, and 14 readers from 9 countries completed the reader study. The readers’ experience in reading mammograms ranged from 1 to 28 years (median of 10.5; mean of 11.8; SD of 8.6).

### 2.7. Statistical Analysis

#### 2.7.1. Discriminative Ability of AI

To assess the AI system’s discriminative ability with respect to all available subsets from which cases for the reader study were drawn, we constructed ROC curves and calculated the associated AUC values, including the 95% confidence intervals using the DeLong method. These were based on the AI scores and true outcomes per case.

Importantly, this ROC analysis was performed on the full available enriched screening datasets from which the reader study cases were selected. By using the entire source datasets, we aimed to provide contextual information on the AI’s performance in those original screening environments. These various datasets originated from different countries, differing in screening setting, target screening population, and cancer prevalence (ranging from 14 to 30%). By using the entire source datasets, we aimed to provide contextual information on the AI’s performance in those original screening environments. This ROC analysis included our full retrospective dataset, separate from the subsequent reader study analyses, which focused exclusively on a purposefully enriched sample of challenging, cancer-positive cases.

Further statistical analysis was performed comparing AI-missed cases and human-missed cases using the reader study results. This involved several assessments to determine the agreement among human readers, and to descriptively analyze their decisions regarding different parameters from the BI-RADS lexicon and from the case-level PoM scores.

For most questions (referring to breast composition, visibility, shape, density, calcifications, and the forced likelihood-of-malignancy 3-point score), categorical and binary answers were applied, allowing for the calculation of inter-rater agreement using Fleiss’ kappa. For the PoM scores, considering 0–100% as semi-continuous values, the inter-rater agreement could be calculated using the intra-class correlation coefficient (ICC).

The degree of inter-rater agreement was evaluated using the following Fleiss’ kappa (k)- and ICC-criteria [28]: <0 reflects ‘poor’, 0 to 0.20 ‘slight’, 0.21 to 0.4 ‘fair’, 0.41 to 0.60 ‘moderate’, 0.61 to 0.8 ‘substantial’, and above 0.81 ‘almost perfect’.

#### 2.7.2. Breast Composition

The breast density category was measured by AI and also estimated by human readers. Descriptive analysis was performed to summarize the distribution of breast density as determined by AI. Inter-rater agreement was assessed to measure the level of agreement among human readers.

The chi-square goodness-of-fit test was applied to assess the agreement between the distribution of breast density scores provided by the AI system and by the human readers.

#### 2.7.3. Lesion Visibility

Inter-rater agreement was calculated to determine the level of agreement among the human readers regarding the presence or absence and type of lesion. The percentage of cases where readers identified a lesion, including the type (architectural distortion, calcifications, mass, no visible lesion) was descriptively analyzed.

Inter-rater agreement was calculated to measure the level of agreement among the readers in determining the degree of visibility of the abnormality and the type of visible lesion.

#### 2.7.4. PoM Scores

Descriptive analysis was performed to summarize the mean and median ratings, including the range and IQR. Inter-rater agreement on case-level PoMs was calculated to determine the level of agreement among the readers.

#### 2.7.5. Visible Mass Characteristics

Inter-rater agreements were calculated to measure the level of agreement among the readers in describing the shape, margins, and density of the mass, separately. The distributions of how the readers scored these three mass characteristics were analyzed descriptively.

#### 2.7.6. Lesion Size

Following the above analyses, a measurement of the identified lesions was conducted for each case by an independent experienced radiologist, blinded to whether cases were human- or AI-missed. This reader reviewed the coordinates where different readers had indicated the location of a lesion for each case, and the measurement was performed on the lesion or suspicious area at the location where the majority of readers reached a consensus, in order to determine its size. The sizes of the lesions in the AI-missed and the human-missed cases were compared by descriptive analyses and the Mann–Whitney U test.

Additionally, the differences in observations between the AI-missed and human-missed cases were tested per outcome category for the categorical data on lesion type and visible mass characteristics using the McNemar test, including adjusted *p*-values after Bonferroni correction. The median differences in PoM scores were tested by the Wilcoxon signed rank test for non-normally distributed data.

Inferential statistical tests were two-tailed, and *p* < 0.05 was considered a significant difference.

All analyses were conducted using R Studio version 2022.12.0.

## 3. Results

### 3.1. Discriminative Ability of AI

**Figure 1:**
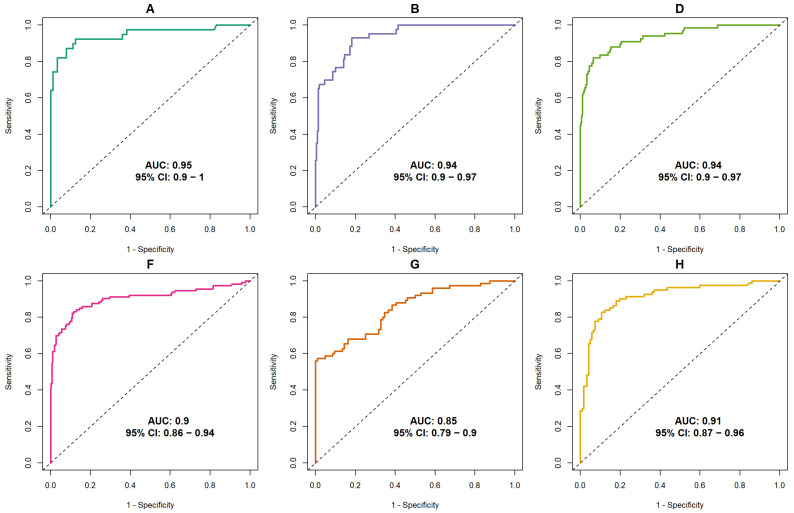
Discriminative ability of AI across the six included subsets (A, B, D, G, F, H; also described in Table 1), expressed by the receiver operating characteristic (ROC) curves and the area under the curve (AUC), including the 95% confidence interval (CI) around the AUC.

ROC analysis resulted in the following areas under the curves (AUCs) for the AI system per subset: 0.946 (95% CI: 0.896–0.996) [A]; 0.936 (95% CI: 0.902–0.971) [B]; 0.936 (95% CI: 0.901–0.970) [D]; 0.903 (95% CI: 0.862–0.944) [F]; 0.847 (95% CI: 0.789–0.905) [G]; and 0.914 (95% CI: 0.872–0.956) [H] (Figure 1).

### 3.2. Selection of Discrepant Cases

Based on the original human multi-reader outcomes (Table 1) and the original case-level truth outcome, we were able to select 34 cases with discrepant outcomes based on human and AI ratings according to this study’s protocol conditions. However, based on the human ratings from the reader study, for two cases, it remained uncertain which laterality included the BC. Therefore, these two cases were excluded from the study. For the remaining four-view cases, we retained the two-view images from the laterality that included the BC based on majority vote.

Due to data corruption, six cases unfortunately had to be excluded. Our final selection involved 15 cases with AI-missed cancer (<10.00% BC suspicion) and 11 cases with human-missed cancer.

### 3.3. Reader Study Results

Table 2 shows the distribution of breast density categories as rated by the AI and the human readers for AI- and human-missed cancer cases, and the characteristics of the missed cancers according to the reader study.

The missed lesion types were similar between the AI- and human-missed cancers, with masses being the most frequently missed lesions in both settings (40.5% (85/210) AI-missed, 41.6% (64/154) human-missed). Calcifications seemed to be more commonly missed among human assessment (18.1% (38/210) AI-missed, 27.3% (42/154) human-missed). A higher fraction of lesions missed by humans compared to AI was rated to be very visible in retrospect (17.1% (36/210) AI-missed, 26.0% (40/154) human-missed).

Masses were similar in shape, but AI-missed cancers had a lower density than human-missed cancers. Margins appeared a bit more frequently indistinct in human-missed cancers (12.4% (26/210) AI-missed, 18.8% (29/154) human-missed).

Typically benign calcifications were seen commonly in both AI-missed and human-missed cancers (44.8% (94/210) AI-missed, 46.8% (72/154 human-missed)). However, typically malignant calcifications were reported more frequently in human-missed cancers (13.8% (29/210) AI-missed, 28.6% (44/172) human-missed).

Based on the PoM scores, the human-missed cancers were more suspicious than the AI-missed cancers (average: 25.8% AI-missed, 33.8% human-missed; median: 15% AI-missed, 30% human-missed), and a higher fraction was scored to be very likely malignant (19.5% (41/210) AI-missed, 32.5% (50/154) human-missed).

Further details regarding the differences between AI-missed and human-missed cases, including lesion type, visibility, PoM, shape, density, margins, calcifications, and likelihood of malignancy, are summarized in Table 2.

#### Lesion Size

Table 3 shows the lesion sizes of the AI- and human-missed cancers. The median lesion size across all views was 11.5 mm. When considering all views combined, lesion sizes in the AI-missed cases were significantly smaller than in the human-missed cancers (median of 9.0 mm and IQR of 6.5–12.0 versus median of 21.0 mm and IQR of 10.5–41.0; *p* = 0.0014).

## 4. Discussion

The main goal of this study was to evaluate whether cancer cases missed by AI and detected by humans or vice versa were different. The results show that the AI-missed human-detected cancers were, in general, somewhat smaller and less suspicious than the human-missed AI-detected cancers. The presence of suspicious calcifications in 28.6% (44/154) of human-missed cancers, the on-average larger size, and the higher suspiciousness scores suggest that the human-missed cancers were more often errors in detection (i.e., overlooked by human readers), whereas the AI-missed cancers were more likely errors in classification, as their suspicion scores were below the recall threshold—sometimes close to the cutoff.

The AI system in our study demonstrated a relatively high discriminative capacity. In all of our subsets, the AUC of this AI system varied between 0.847 and 0.946 using a version of AI developed in 2023. These values are above the average AUC of 0.840 reported in the study conducted by Rodriguez-Ruiz et al. (2019), which used an AI system developed in 2017 and included a partially overlapping dataset. Considering the ongoing advancements in AI with regard to detection of breast cancer in mammograms, it is to be expected that AI technology, and hence, its discriminative capacity, will continue to improve.

However, while we recognize the achievements of AI in breast screening, to the point where it now closely rivals human reading [10], our study shows that it does not always select the same cases as human radiologists as suspicious. While unconscious biases in humans can affect breast imaging interpretations [29], suggesting that AI may be less susceptible to these errors, it is crucial to acknowledge that AI is not free from its own biases. Nonetheless, a natural consequence of the high performance of the AI system on this dataset is that relatively few discrepant cases remain for in-depth study and analysis regarding the radiologic features of missed lesions. Considering that cancer detection rates in mammography screening are only in the order of 4–6 per 1000 cases, and most cases are detected by both human readers and AI, the 26 discrepant cases represent a screening set of multiple thousands of screening cases.

The results of our reader study (Table 2) demonstrate that the AI-missed cases were more frequently classified as having “no visible lesion” compared to the human-missed cases. The visibility assessments for both the AI- and human-missed cases were often rated as “slight” or “not applicable” (due to no visibility), with low inter-rater agreement.

In contrast, the human-missed cases were more often rated as ‘very likely’ (forced ordinal scale) to be malignant and received higher median and mean PoM scores compared to the selected AI-missed cases. However, the slight to poor inter-rater agreement (ICC of 0.15 and kappa of 0.05) reveals the high degree of heterogeneity with which these human-missed cases were assessed.

The readers were aware that the cases included in the reader study most likely involved challenging cases in which previous AI and human scores did not align. This suggests that assessments were likely conducted with less objectivity, possibly leading the readers to be inclined to give BC-positive scores. However, the readers were not aware whether a case was missed by AI or by a human reader, and therefore, the relative difference observed is likely real.

It was notable that regarding the likelihood of malignancy, the proportion of ratings labeled as “uncertain”, was similar and also relatively high for both groups (41.4% (87/210) and 40.3% (62/154) for AI- and human-missed cases, respectively) affirming that the selected cases were overall challenging to interpret.

The notably low inter-rater-agreement for human-missed cases regarding malignancy, which improved for the AI-missed cases, suggests that human-missed cases were more challenging, while AI-missed diagnoses may not necessarily pose the same level of interpretation difficulties for human readers.

Our single-reader assessment suggests that the AI-missed cases involved significantly smaller lesions than the human-missed cases. This observation may align with the slightly higher fraction of calcifications in the human-missed cases. This could mean that radiologists may categorize suspicious areas of calcifications as benign more often than AI.

Very dense breasts do not appear to be a standalone feature that makes cases challenging to interpret. Most of the selected cases involved density categories B, C, and D. It is worth noting that only density category A breasts were very rare among the selected discrepant cases. From our reader study, only 11% (24/210) of density ratings for the AI-missed cases were classified as density category A, which is similar to the population average. However, there were no density A ratings within the human-missed cases. The fact that the distribution across density categories was less skewed for the AI-missed cases compared to the human-missed cases suggests that breast density may have a slightly smaller impact on the likelihood of missing a breast cancer diagnosis for AI in comparison to human readers and AI might have slightly better performance on dense breasts.

We acknowledge the limitations of using a standard PC monitor and the small number of discrepant cancer cases. The absence of laterality and lesion-specific ground truth thereby limited more detailed analyses of cancer visibility. However, this does not detract from the value of our study, which serves as an exploratory opportunity to examine discrepancies between AI and human readings.

The available multi-center data from which discrepant cases were selected, as well as the cases that were actually selected for the reader study, originated from six centers. However, it appeared that the selected cases from the Padua dataset were overrepresented in the final selection (12/26 (46%) of the cases), including 10 out of 11 human-missed cases. Although this subset was one of the larger subsets, it was not the largest, it did not have the most readers, nor was the average recall rate per reader notably lower. Therefore, it is challenging to pinpoint why there were more instances of discrepant human–AI screening outcomes in these cases. The substantial presence of Padua cases in the reader study might reduce the multi-center nature of the data. However, achieving a more proportional selection of discrepant cases per subset was not feasible in this study due to the scarcity of discrepant cases.

As ground-truth outcomes were only available at the case level, localization was, by definition, based on majority vote in the reader study. There is always a risk that the majority of readers pointed out a mammography finding that was not later confirmed as cancer. This is particularly true for the four-view mammography exams. Eventually, six cases were excluded from subsequent analysis because <60% of the ratings agreed on the lesion location, reducing the study sample. However, we believe this was an important step, as it prevented erroneously describing lesion features.

In conclusion, lesions in all missed cases were often described as having ‘low visibility’, ‘indistinct margins’, or an ‘irregular shape’. The overall low inter-rater agreement on the items assessed during the reader study suggests that the interpretation of the selected images was challenging. Due to the overall low inter-rater agreement scores, it is challenging to identify specific radiological features that describe the selected discrepant cases. However, the findings do suggest slight variations among cases with missed diagnoses by AI versus human readers. The results suggest that lesions missed by AI more frequently involve relatively small and less obvious masses, while lesions missed by human radiologists tend to involve larger suspicious areas where the presence of calcifications may inaccurately be regarded as a benign condition. While definitive conclusions are premature, the findings highlight the complementary roles of AI and human readers in mammographic interpretation.

## Figures and Tables

**Table 1:** Details of each dataset collected for this study. Abbreviations: GE = GE HealthCare (General Electric Company; Hoevelaken, Netherlands); Sectra = Sectra AB (Teknikringen, Sweden), Hologic = Hologic Inc. (Marlborough, MA, United States), Siemens = Siemens AG (München, Germany); BI-RADS = Breast Imaging Reporting and Data System; PoM = probability of malignancy; N = number; Screening = screening exams obtained from a population-based screening program (low-risk screening populations); Clinical = screening exams obtained from a clinical screening setting in a hospital (usually higher-risk populations). More details can be found in the respective publications: A [19]; B [20]; D [18]; F [21]; G [22]; H [23].

Dataset (Year)	Vendor (s)	Population	Exam Type	Total Exams	Cancer, n (%)	Benign Lesion	Normal	Readers	Experience (Years)	Mean Recall Rate (%) Human Readers	Scoring Scale
A (2011)	GE Sectra	40–80 (mean of 56) years, Screening (86), Clinical (43)	Bilateral no priors	129	40 (31)	23	66	14	3–25 (mean of 10)	60	BI-RADS
B (2012)	GE	51–86 (mean of 60) years, Screening	Bilateral + priors	263	43 (16)	110	110	6	1–34	38	PoM
D (2013)	GE	>40 years, 50–74 years, Screening + Clinical	Unilateral + priors	469	68 (15)	200	201	6	5–30	43	BI-RADS
F (2017)	Hologic	34–92 (mean of 55) y, Screening + Clinical	Bilateral + priors	585	113 (19)	160	313	3	10–20	41	BI-RADS
G (2017)	Siemens	30–88 (mean of 52) years, Screening + Clinical	Unilateral + priors	179	75 (42)	49	55	6	3–44 (mean of 22)	47	PoM BI-RADS
H (2018)	Siemens	36–84 (mean of 56) years, Screening + Clinical	Bilateral + priors	204	82 (40)	43	80	4	>5	49	BI-RADS

**Table 2:** Inter-rater agreement, descriptive, and inferential results of assessed items in the reader study. AI-Missed: Cases categorized as AI-missed cancer if breast cancer was diagnosed, at least one human reader in the retrospective studies rated the case as BI-RADS > 2, and the AI rated it as <10.00% breast cancer suspicion. Human-Missed: Cases categorized as human-reader-missed cancer if breast cancer was diagnosed, at least one human reader during the retrospective studies scored the case as BI-RADS < 3, and the AI rated it as ≥10.00%. Observations: During the reader study, each of the assessed AI-missed (n = 15) and human-missed (n = 11) lesions was evaluated once by each of the 14 readers. For the categorical variables, the percentage (%) of these observations falling into specific categories is presented. For the continuous variables, corresponding statistical measures are expressed: average, standard deviation (SD), median, interquartile range (IQR), minimum (Min), and maximum (Max). Abbreviations: Adj. *p* = Adjusted *p* value after Bonferroni correction. Kappa = Inter-rater agreement expressed by Fleiss’ Kappa, applicable to binary and categorical data. ICC = Inter-rater agreement expressed by the intraclass correlation coefficient, applicable to (semi)-continuous data. Inter-Rater Agreement Criteria: <0 reflects ‘poor’; 0 to 0.20, ‘slight’; 0.21 to 0.4, ‘fair’; 0.41 to 0.60, ‘moderate’; 0.61 to 0.8, ‘substantial’; and above 0.81, ‘almost perfect’.

Lesion Type	AI-Missed (n = 15)		Human-Missed (n = 11)		*p*-Value
Breast Density	AI-Scored Observations, n (%)	Human-Scored Observations, n (%)	AI-Scored Observations, n (%)	Human-Scored Observations, n (%)	
A	2 (13)	24/210 (11)	0 (-)	0/154 (-)	
B	8 (53)	98/210 (47)	3 (27)	43/154 (28)	
C	3 (20)	67/210 (32)	4 (36)	62/154 (40)	
D	2 (13)	21/210 (10)	4 (36)	49/154 (32)	
Fleiss’ Kappa (human-scored breast density)	0.40 (95%CI: 0.36–0.44; *p* < 0.0001)		0.47 (95%CI: 0.43–0.52; *p* < 0.0001)		
Lesion Type	Observations		Observations		
Architectural distortion	26/210 (12.4)		24/154 (15.6)		≈1 Adj. *p* ≈ 1
Calcifications	38/210 (18.1)		42/154 (27.3)		0.396 Adj. *p* ≈ 1
Mass	85/210 (40.5)		64/154 (41.6)		0.211 Adj. *p* = 0.845
No visible lesion	61/210 (29.0)		24/154 (15.6)		0.001 Adj. *p* = 0.003
Kappa (lesion type)	0.27 (95%CI: 0.24–0.29; *p* < 0.0001)		0.26 (95%CI: 0.24–0.28; *p* < 0.0001)		
Lesion Visibility	Observations		Observations		
Slight	56/210 (26.7)		42/154 (27.3)		0.185 Adj. *p* = 0.738
Moderate	51/210 (24.3)		45/154 (29.2)		0.598 Adj. *p* ≈ 1
Very	36/210 (17.1)		40/154 (26.0)		≈1 Adj. *p* ≈ 1
Not Applicable	67/210 (31.9)		27/154 (17.5)		<0.001 Adj. *p* < 0.001
Kappa (visibility)	0.12 (95%CI: 0.107–0.129; *p* < 0.0001)		0.15 (0.95%CI: 0.14–0.16; *p* < 0.0001)		
Probability of Malignancy (PoM)	Observations		Observations		
Average	25.8%		33.8%		
SD	27.0%		25.8%		
Median	15.0%		30.0%		0.002
IQR	35.0%		40.0%		
Min	0.0%		0.0%		
Max	100.0%		96.0%		
ICC	0.40 (95%CI: 0.24–0.64; *p* < 0.0001)		0.15 (95%CI: 0.05–0.41; *p* < 0.001)		
Lesion Shape	Observations		Observations		
Round	27/210 (12.8)		17/154 (11.0)		0.522 Adj. *p* ≈ 1
Oval	16/210 (7.6)		16/154 (10.4)		1.000 Adj. *p* ≈ 1
Irregular	49/210 (23.3)		40/154 (26.0)		0.456 Adj. *p* ≈ 1
Not Applicable	118/210 (56.2)		81/154 (52.6)		0.002 Adj. *p* ≈ 0.006
Kappa (shape)	0.27 (95%CI: 0.25-0.30; *p* < 0.0001)		0.18 (95%CI: 0.16–0.19; *p* < 0.0001)		
Lesion Density	Observations		Observations		
Fat	1/210 (0.5)		1/154 (0.6)		1.000 Adj. *p* ≈ 1
Low	10/210 (4.8)		5/154 (3.2)		0.423 Adj. *p* ≈ 1
Equal	68/210 (32.4)		32/154 (20.8)		0.002 Adj. *p* = 0.009
High	31/210 (14.8)		44/154 (28.6)		0.166 Adj. *p* = 0.829
Not Applicable	100/210 (47.6)		72/154 (46.8)		0.007 Adj. *p* = 0.037
Kappa (lesion density)	0.20 (0.95%CI: 0.18, 0.21; *p* < 0.0001)		0.26 (0.95%CI: 0.23–0.28; *p* < 0.0001)		
Margins of a Visible Mass	Observations		Observations		
Circumscribed	19/210 (9.0)		12/154 (7.8)		0.845 Adj. *p* ≈ 1
Obscured	14/210 (6.7)		14/154 (9.1)		1.000 Adj. *p* ≈ 1
Microlobulated	13/210 (6.2)		5/154 (3.2)		0.099 Adj. *p* = 0.594
Indistinct	26/210 (12.4)		29/154 (18.8)		0.784 Adj. *p* ≈ 1
Spiculated	26/210 (12.4)		16/154 (10.4)		0.165 Adj. *p* = 0.989
Not Applicable	112/210 (53.3)		78/154 (50.6)		0.003 Adj. *p* ≈ 1
Kappa (margins)	0.29 (95%CI: 0.26–0.31; *p* < 0.0001)		0.20 (0.95%CI: 0.18–0.21; *p* < 0.0001)		
Presence and Type of Calcifications	Observations		Observations		
None	87/210 (41.4)		38/154 (25.0)		<0.001 Adj. *p* < 0.001
Typically benign	94/210 (44.8)		72/154 (46.8)		0.091 Adj. *p* = 0.272
Typically suspected of malignancy	29/210 (13.8)		44/154 (28.6)		0.382 Adj. *p* ≈ 1
Kappa (calcifications)	0.30 (95%CI: 0.27–0.32; *p* < 0.0001)		0.22 (0.95%CI: 0.20–0.25; *p* < 0.0001)		
Likelihood of Malignancy	Observations		Observations		
Unlikely	61/210 (29.0)		29/154 (18.8)		0.003 Adj. *p* = 0.012
Uncertain	87/210 (41.4)		62/154 (40.3)		0.068 Adj. *p* = 0.271
Very likely	41/210 (19.5)		50/154 (32.5)		0.828 Adj. *p* ≈ 1
Not Applicable	21/210 (10.0)		13/154 (8.4)		0.137 Adj. *p* = 0.550
Kappa (likelihood of malignancy)	0.16 (95%CI: 0.15–0.18; *p* < 0.0001)		0.05 (95%CI: 0.04–0.05; *p* = 0.016)		

**Table 3:** Single-Reader Assessed Lesion Sizes: Lesion sizes are summarized using descriptive statistical measures for continuous data: median, mean, standard deviation (SD), minimum (Min), maximum (Max), and interquartile range (IQR). AI-Missed: Cases categorized as AI-missed cancer if breast cancer was diagnosed, at least one human reader in the retrospective studies rated the case as BI-RADS > 2, and the AI rated it as <10.00% breast cancer suspicion. Human-Missed: Cases categorized as human-reader-missed cancer if breast cancer was diagnosed, at least one human reader during the retrospective studies scored the case as BI-RADS < 3, and the AI rated it as ≥10.00%. Abbreviations: CC = craniocaudal; MLO = mediolateral oblique; mm = millimeters.

Subset		Lesion Size [mm]				
	Median	Mean	SD	Min.	Max.	IQR
All CC views	12.0	16.9	14.6	3.0	53.0	8.0–19.0
All MLO views	11.0	16.5	15.7	2.4	64.0	7.0–16.0
All views combined	11.5	16.7	15.0	2.4	64.0	8.0–18.8
AI-missed CC views	9.5	9.7	4.9	3.0	19.0	6.5–12.0
AI-missed MLO views	9.0	9.8	5.0	2.4	21.0	7.5–12.0
All AI-missed views combined	9.0	9.7	4.9	2.4	21.0	6.5–12.0
Human-missed CC views	21.0	25.9	17.9	4.0	53.0	12.0–39.5
Human-missed MLO views	22.0	26.5	20.8	4.0	64.0	9.0–43.0
All human-missed views combined	21.0	26.2	19.3	4.0	64.0	10.5–41.0

## Data Availability

The data presented in this study are available on request from the corresponding author.

## Supplementary Materials

The following supporting information can be downloaded at https://www.mdpi.com/article/10.3390/diagnostics15121566/s1.
