# Supplementary material for: An Exploration of Discrepant Recalls Between AI and Human Readers of Malignant Lesions in Digital Mammography Screening
Source: Diagnostics (Basel). 2025 Jun 19;15(12):1566. doi: 10.3390/diagnostics15121566 (PMC12191860; doi:10.3390/diagnostics15121566)

diagnostics

MDPI

Supplement 1: Questions as Presented in Online Reader Study

How would you describe the breast composition?*

- The breasts are almost entirely fatty
- There are scattered areas of fibroglandular density
- The breasts are heterogeneously dense, which may obscure small masses
- The breasts are extremely dense, which lowers the sensitivity of mammography

Is there a lesion present in this case?*

- Yes: architectural distortion
- Yes: calcifications
- Yes: mass
- No: no visible lesion

Select the most suitable answere. If there is no visible lesion, please choose “inapplicable” for the remaining questions. In case of multiple lesions: please choose the answer that is most representative of the presented images.

How visible is/are the lesion(s) on these images?*

- slightly visible
- moderately visible
- very visible
- inapplicable

Please indicate where in the image the lesion(s) are located

[….]

If there is no visible lesion: you can skip this question. If the lesion is only visible on the second view: please leave this question open and annotate the location of the lesion in the next question.

Please indicate where in the image the lesion(s) are located

[….]

If there is no visible lesion: you can skip this question.

How would you score the probability of malignancy (%) within this case?*

Please choose a value between 0 and 100 (%)

Describe the shape of the visible mass(es):*

- Round
- Oval
- Irregular
- Inapplicable

If the lesion is suspected to be a mass: describe the mass(es) by answering the question. Otherwise: choose "inapplicable".

Describe the margins of the visible mass(es):*

- Circumscribed
- Obscured
- Microlobulated
- Indistinct
- Spiculated
- Inapplicable

If the lesion is suspected to be a mass: describe the mass(es) by answering the question. Otherwise: choose "inapplicable"

Describe the density of the visible mass(es):*

- Fat
- Low
- Equal
- High
- Inapplicable

If the lesion is suspected to be a mass: describe the mass(es) by answering the question. Otherwise: choose "inapplicable".

How would you describe the presence of calcifications?*

- None
- Typically benign
- Typically suspected of malignancy

In case of a visible lesion, how would you classify the likelihood of a malignancy in this case?*

- Unlikely
- Uncertain
- Very likely
- Inapplicable

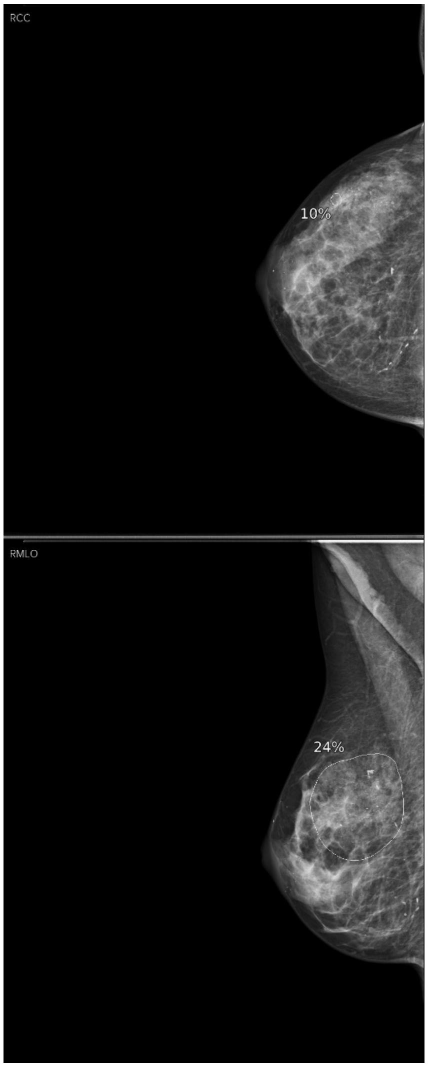

Example 1: *Human-missed breast cancer positive mammogram (Cranio-Caudal right and Medio-Oblique right): Scored positive by AI (maximum score of right laterality 24.00 %) and missed by 1 out of 4 original human readers (BI-RADS 2, with no visual information available). In our online reader study, 11 out of 14 readers indicated the location of the suspected lesion in the image (only indicated if visible). These annotations are displayed in the figure below.*

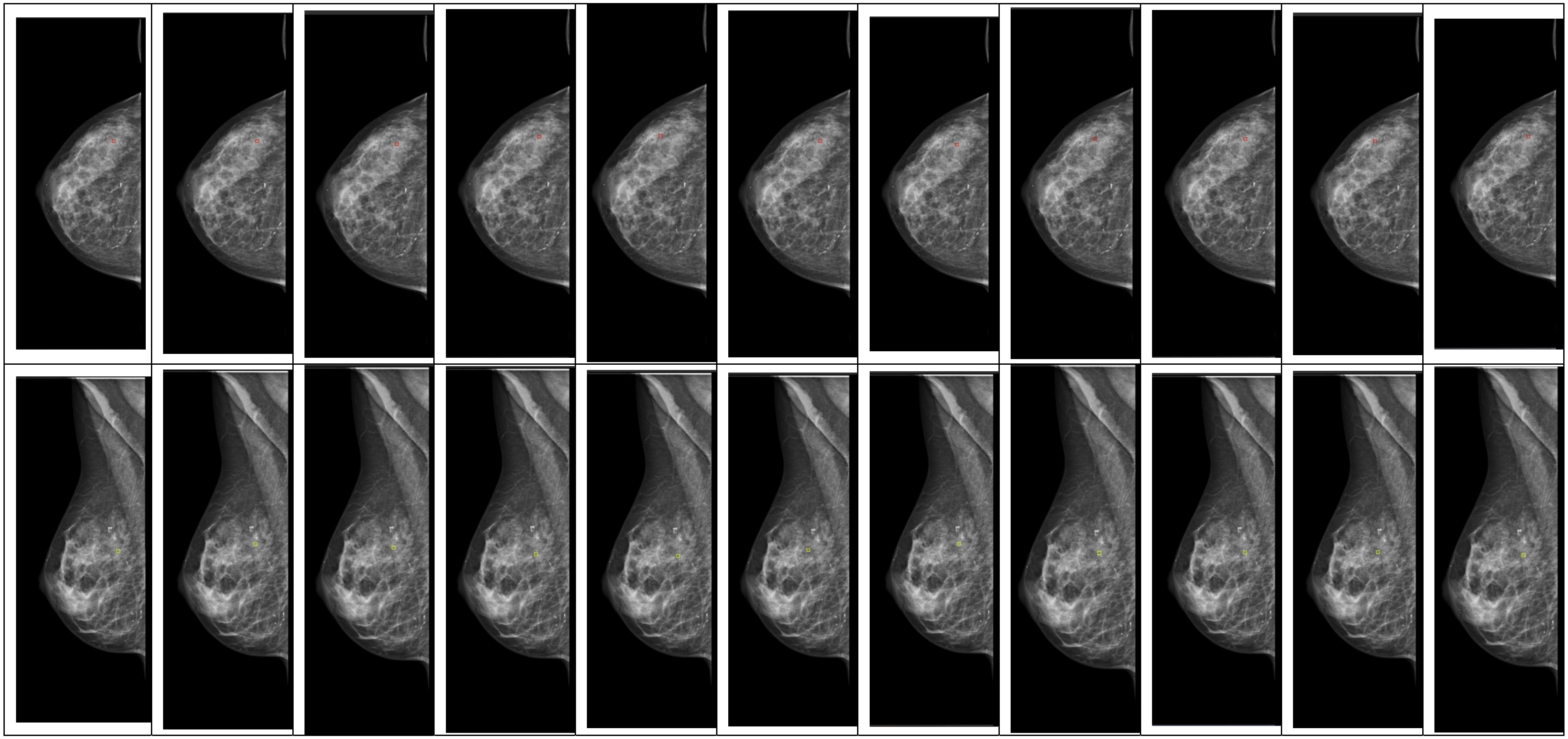

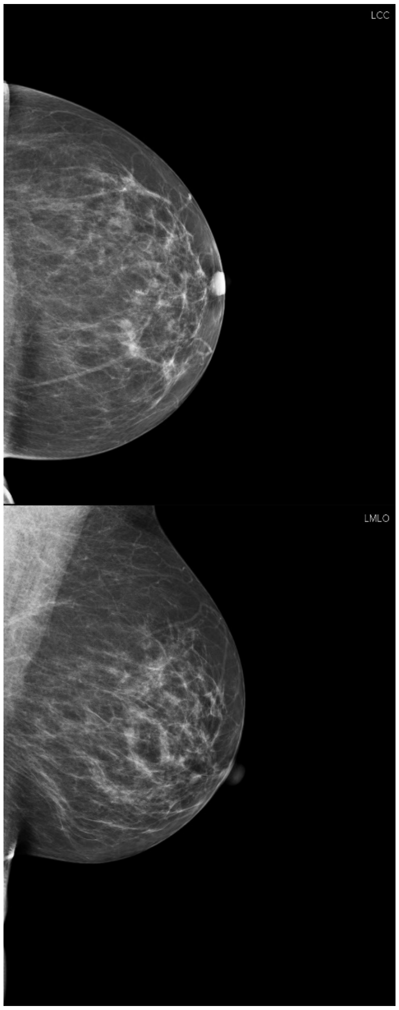

Example 2: *AI-missed breast cancer on a positive mammogram (Cranio-Caudal left and Medio-Oblique left): Scored as negative by AI (5.39%, with no visual information available due to a score below 10.00%) and identified by 2 out of 6 original human readers (PoM 50% and 54%, with no visual information available). In our online reader study, 11 out of 14 readers indicated the location of the suspected lesion in the image (only indicated if visible). These annotations are displayed in the figure below.*

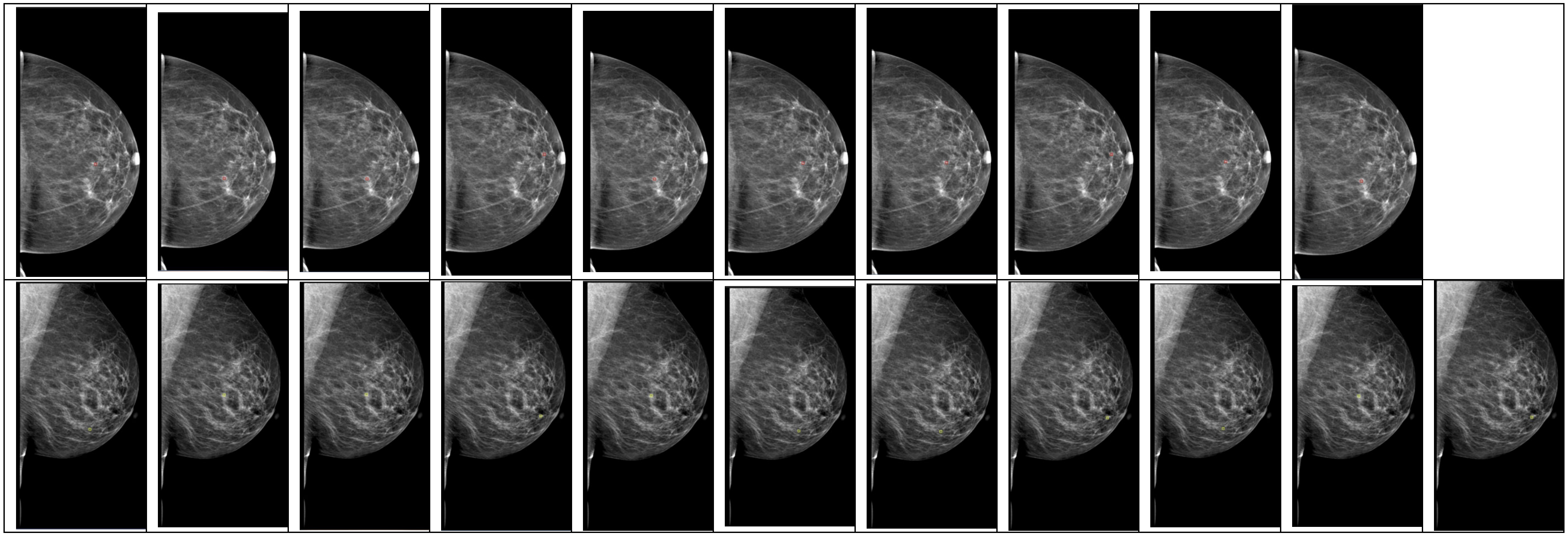

Supplement: Supplementary file 1 [file diagnostics-15-01566-s001.zip › diagnostics-3618851-supplementary.pdf]
